# Radiotherapy in patients with NSCLC developing progressive disease during immune checkpoint inhibition: Abscopal responses and survival

**DOI:** 10.1016/j.ctro.2026.101125

**Published:** 2026-02-11

**Authors:** Justus Kaufmann, Maike Trommer, Alexander Rühle, Allison Lamrani, Charlotte Frei, Matthias Mäurer, Georg Wurschi, Ping Jiang, Felix Ehret, Andrea Baehr, Annika Hardt, Raphael Bodensohn, Lukas Käsmann, Maria Waltenberger, Julian P. Layer, Davide Scafa, Esther G.C. Troost, Sally A. Elkhamisy, Danny Jazmati, Ilinca Popp, Sebastian Neppl, Anna Hagemeier, Simone Ferdinandus

**Affiliations:** a University Medical Center of the Johannes Gutenberg University, Department of Radiation Oncology, Mainz, Germany; b University Hospital Bonn, Department of Radiation Oncology, Bonn, Germany; c Center for Integrated Oncology Aachen Bonn Cologne Duesseldorf (CIO ABCD), Cologne, Germany; d Olivia Newton-John Cancer Wellness & Research Centre, Austin Health, Department of Radiation Oncology, Melbourne, Australia; e University of Cologne, Center for Molecular Medicine Cologne (CMMC), Cologne, Germany; f Medical Center, Faculty of Medicine, University of Freiburg, Department of Radiation Oncology, Freiburg, Germany; g University of Leipzig Medical Center, Department of Radiation Oncology, Leipzig, Germany; h University Hospital Erlangen, Department of Radiation Oncology, Erlangen, Germany; i University Hospital Jena, Department of Radiation Oncology, Jena, Germany; j University Hospital Jena, Clinician Scientist Program OrganAge, Jena, Germany; k University Hospital Jena, Clinician Scientist Program, IZKF, Jena, Germany; l Clinic for Radiation Oncology and Radiotherapy, Lüdenscheid Clinic, Lüdenscheid, Germany; m Charité – Universitätsmedizin Berlin, Corporate Member of Freie Universität Berlin and Humboldt-Universität zu Berlin, Department of Radiation Oncology, Berlin, Germany; n German Cancer Consortium (DKTK), partner site Berlin, a partnership between DKFZ and Charité – Universitätsmedizin Berlin, Germany; o University Medical Hospital, Hamburg-Eppendorf, Department of Radiation Oncology, Hamburg, Germany; p Outpatient Center of the University Medical Hospital Hamburg-Eppendorf, Department of Radiotherapy and Radiation Oncology, Hamburg, Germany; q University Hospital, LMU Munich, Department of Radiation Oncology, Munich, Germany; r University Hospital Tübingen, Department of Radiation Oncology, Tübingen, Germany; s Department of Radiation Oncology, Klinikum rechts der Isar, Technical University of Munich, Munich, Germany; t Hospital of Bolzano, Department of Radiation Oncology, (SABES-ASDAA), Bolzano-Bozen, Italy; u Teaching Hospital of Paracelsus Medical University Salzburg, Salzburg, Austria; v University Hospital Bonn, Institute of Experimental Oncology, Bonn, Germany; w OncoRay – National Center for Radiation Research in Oncology, Faculty of Medicine and University Hospital Carl Gustav Carus, TUD Dresden University of Technology, and Helmholtz-Zentrum Dresden-Rossendorf, Dresden, Germany; x National Center for Tumor Diseases (NCT/UCC), Dresden, Germany; y Faculty of Medicine and University Hospital Carl Gustav Carus, TUD Dresden University of Technology, Department of Radiotherapy and Radiation Oncology, Dresden, Germany; z University Hospital Dusseldorf, Medical Faculty, Heinrich-Heine-University Dusseldorf, Department of Radiation Oncology, Dusseldorf, Germany; aa Faculty of Medicine and University Hospital Cologne, Department of Radiation Oncology, Cyberknife and Radiotherapy, Cologne, Germany; ab Institute of Medical Statistics and Computational Biology, Medical Faculty and University Hospital, University of Cologne, Cologne, Germany

**Keywords:** Non-small cell lung cancer, NSCLC, Immunotherapy, Radiotherapy, Abscopal effect

## Abstract

**Objective:**

Increasing evidence suggests that RT and ICI have synergistic effects on anti-tumor immune response, and abscopal responses (AR) have been described more frequently with this combination. Here, we analyzed the frequency and factors associated with AR in NSCLC patients receiving ICI and RT.

**Methods:**

In ARTIC (ARO 2022–10, DRKS00032390), a multicenter, retrospective study, patients treated with RT for progressive disease during or after receiving ICI were analyzed for abscopal benefit (AB) and overall survival (OS). Patients were grouped by non-irradiated lesion response; logistic and Cox regression were used to analyze factors linked to AR, AB, and OS.

**Results:**

Fifty-six patients from 13 centers with 137 non-irradiated lesions were analyzed. Most received hypofractionated or stereotactic RT. The most common RT sites were lung, bone, and brain. Thirty-three patients showed AB, with 15 patients developing an AR in at least one non-irradiated lesion. In the logistic regression analysis, older age was associated with a higher rate of AP (odds ratio (OR): 1.07, 95%-CI: 1.00–1.14; p = 0.05). Male sex showed a trend towards higher risk of AP (OR = 3.13; 95%- CI: 0.93–11.86; p = 0.075). In the multivariable Cox analysis, ECOG performance score at the start of RT (hazard ratio (HR): 0.29, 95%-CI: 0.12–0.69; p < 0.01) and oligometastatic disease (HR: 0.34, 95%-CI: 0.12–0.94; p = 0.039) were significantly associated with improved OS.

**Conclusion:**

RT was able to induce AR in some NSCLC patients who were progressive under ICI, possibly delaying a switch in systemic treatment. AB was less frequent in older patients and in men.

## Highlights

• RT induced abscopal responses in 27% of progressive NSCLC patients.
• Abscopal benefit was less common in older patients and in men.
• Better ECOG and oligometastatic status predicted longer survival.
• Local RT may delay systemic therapy switch during ICI progression.

## Introduction

Non-small cell lung cancer (NSCLC) is considered immunogenic and demonstrates significant and clinically meaningful responses to treatment with immune checkpoint inhibitors (ICI) [1]. Several large randomized trials have shown the efficacy of ICI in both curative and palliative settings [2], [3], [4], [5], [6]. In addition, the combination of radiotherapy (RT) and ICI has been shown to improve the outcome of NSCLC patients in a large prospective and randomized trial [7], [8]. Several preclinical and clinical studies have also demonstrated this synergistic effect, not only for local tumor control but also for systemic treatment efficacy [9], [10], [11].

The increased systemic treatment efficacy can result in so-called abscopal effects (AbE), which are defined as regression of non-irradiated (tumor) lesions (NILs) following RT [11], [12]. These effects have been described sporadically for several years and are hypothesized to occur due to the activation of the anti-tumor immune response after RT of a different lesion [13], [14], [15]. This activation of the anti-tumor immune response is attributed to increased amounts of tumor antigens resulting from radiogenic cell death on one side and reduced immunosuppression due to ICI on the other [16], [17], [18]. Preclinical and recent clinical data support the hypothesis that synergistic effects on NILs exist, with ultrahypofractionated radiotherapy (>5 Gy/fraction) enhancing anti-PD-1 efficacy [10], [19], [20]. With the advent of ICI, AbE have been observed more frequently. However, the actual rate of AbE has not been systematically assessed, and the exact mechanisms of AbE remain elusive. Here, we describe the NSCLC subgroup of a nationwide retrospective study assessing the occurrence and pattern of AbE in a multicentre real-world cohort of metastatic cancer patients with NSCLC.

## Patients and methods.

### Screening and assessment

The study design as well as screening and assessment methods have been published previously [21], [22], [23]. In short, we included NSCLC patients treated between 06/2015 and 06/2021 who had radiologically confirmed tumor progression under ICI (PD-L1/PD-1-inhibitors). Progression was not required to be present in all lesions; inclusion required at least one metastatic lesion not treated with RT and the availability of sufficient imaging for longitudinal assessment.. ICI was initiated at least four weeks prior to RT and continuously applied during the analysis time (RT + 180 days) or discontinued before RT. Any switch to another systemic treatment due to tumor progression (between the time point of progression and the beginning of RT) and lack of cross-sectional imaging data (CT/MRI/PET) were exclusion criteria. This is a subgroup analysis focused on patients diagnosed with NSCLC.

RT was defined as follows: >2 to < 5 Gy = hypofractionated; ≥5 Gy = stereotactic RT; ≤2 Gy = normofractionated [24], [25]. Oligometastatic disease was defined according to the definition proposed by the EORTC, meaning no more than 5 total lesions in no more than 3 different organs [26]. Survival was defined as the time from the end of RT until an event. If no event occurred, patients were censored at the last follow-up.

Non-irradiated lesions (NILs) were assessed using iRECIST criteria, and abscopal responses (AR) were classified accordingly [27], [28]. For response assessment, NILs were measured at multiple time points: pre-ICI (baseline), during ICI (progression under ICI), and after RT (7–180 days post-RT). Lesions showing at least 30% decrease in diameter were classified as AR, while those with at least 20% increase were classified as Abscopal Progression (AP). Lesions with a change between these thresholds were considered stable (Abscopal Control; AC). At the patient level, we defined four response categories: patients with all NILs showing AR, patients with at least one AR, patients with only AC, and patients with at least one AP. To distinguish clinically meaningful responses, the Abscopal Benefit (AB) group was defined as either “AR”, “at least one AR” or “AC”, because disease stabilization in this setting was considered beneficial. Patients who had AP in any NIL were classified as having no AB.

### Statistical analysis

First, descriptive analyses were performed to provide an overview of the study population. Categorical variables are presented as absolute and relative frequencies (percentages), while continuous variables are reported as mean with standard deviation and median with interquartile range. Additionally, a binomial logistic regression analysis was performed to determine whether factors that differed between patients with and without AB were independent predictors of an abscopal response. Due to the small number of events, we limited the analysis to the two factors that had demonstrated the biggest difference between groups, age and sex. As half of the patients had insufficiently recorded laboratory values, the relative lymphocyte count was not included in the analysis. Survival data were plotted using Kaplan-Meier curves. Potential clinical factors influencing OS were subjected to univariable analysis using a log-rank test. Factors significantly associated with OS in univariable analysis were chosen for multivariable analysis. Initial variables used for testing in the multivariable Cox proportional hazards model were age at the start of RT, Eastern Cooperative Oncology Group (ECOG) performance score, oligometastatic disease, PTV size, AB, and location of irradiated lesion. The final model was chosen using a forward stepwise selection process, always including age at the start of RT. To adjust for possible immortal time bias regarding the effect of AB, we repeated the analysis, defining OS as the time between the final imaging and the last follow-up or death. Hazard ratios are displayed, along with their 95% confidence intervals. The proportional hazards assumption was tested and confirmed using Schoenfeld residuals and the global Schoenfeld test. A p-value of < 0.05 was considered statistically significant, although all p-values should be interpreted as exploratory. Statistical analyses were conducted using R Version 4.4.0 [29], [30].

The study was registered in the German working group for radiation oncology (ARO, trial number: ARO 2022–10) and in the German Clinical Trials Register.

## Results

### Patient characteristics

3773 cases from 13 centers were screened, identifying 56 NSCLC patients who progressed during ICI treatment. The mean age ± standard deviation (SD) at initial diagnosis was 62 ± 10.1 years and 64.1 ± 10.4 years when starting RT. 62.5% (35/56) of patients were male. ECOG performance status was distributed as follows: 39% had ECOG 0, 41% had ECOG 1, 12.5% had ECOG 2, 5.5% had ECOG 3, and 2% had ECOG 4. The median LDH level before ICI initiation was 211 U/L (IQR: 180 – 305 U/L). The average body mass index (BMI) ± SD at the beginning of RT was 24.15 ± 4.4 kg/m^2^. The most commonly administered ICI agent was pembrolizumab (30/56, 53.5%), followed by nivolumab (22/56, 39%). The median time between start of ICI and start of RT was 8 months (95% confidence interval (CI)I: 5.2 – 9.2 months). After RT, ICI was continued for a median time of 3.5 months (95-CI: 1.6 – 11 months). Prior to the analyzed RT series, 39% of patients (22/56) had not undergone any RT. A detailed overview of patient characteristics is summarized in Table 1.

**Table 1:** Patient characteristics.

Factor	Levels	AP & Other (no benefit) (N = 23)	AR, AC & min. 1AR (benefit) (N = 33)	Total
Age at RT	Mean (SD)	67.7 (8.6)	61.5 (10.8)	64.1 (10.4)
	Median (IQR)	69 (63.5 – 73.5)	63 (51 – 58)	65 (58 – 71.2)
Sex	Female	5 (21.7)	16 (48.5)	21 (37.5)
	Male	18 (78.3)	17 (51.5)	35 (62.5)
BMI	Mean (SD)	24.1 (4.3)	24.2 (4.6)	24.2 (4.4)
	Median (IQR)	23.9 (21.5 to 27.2)	24.8 (21.3 to 26.0)	24.0 (21.1 to 26.8)
Initial UICC	Stage I	2 (8.7)	1 (3.0)	3 (5.4)
	Stage II	1 (4.3)	0 (0.0)	1 (1.8)
	Stage III	2 (8.7)	3 (9.1)	5 (8.9)
	Stage IV	15 (65.2)	28 (84.8)	43 (76.8)
	(Missing)	3 (13.0)	1 (3.0)	4 (7.1)
ECOG	0	9 (39.1)	13 (39.4)	22 (39.3)
	1	7 (30.4)	16 (48.5)	23 (41.1)
	2	5 (21.7)	2 (6.1)	7 (12.5)
	3	2 (8.7)	1 (3.0)	3 (5.4)
	4	0 (0.0)	1 (3.0)	1 (1.8)
Smoker	smoker	17 (73.9)	27 (81.8)	44 (78.6)
	never smoker	2 (8.7)	4 (12.1)	6 (10.7)
	(Missing)	4 (17.4)	2 (6.1)	6 (10.7)
TPS	<20%	10 (43.5)	11 (33.3)	21 (37.5)
	>= 20%	11 (47.8)	17 (51.5)	28 (50.0)
ICI Agent	Atezolizumab	1 (4.5)		1 (1.9)
	Nivolumab	9 (40.9)	13 (40.6)	22 (40.7)
	Pembrolizumab	11 (50.0)	19 (59.4)	30 (55.6)
	Tislelizumab	1 (4.5)		1 (1.9)
Prior RT	No	10 (43.5)	12 (36.4)	22 (39.3)
	Yes	10 (43.5)	21 (63.6)	31 (55.4)
	(Missing)	3 (13.0)	0 (0.0)	3 (5.4)
RT	hypofractionated	12 (52.2)	18(54.5)	30 (53.6)
	Normofractionated	2 (8.7)	2 (6.1)	4 (7.1)
	stereotactic	9 (39.1)	13 (39.4)	22 (39.3)
CRP	<5	1 (4.3)	8 (24.2)	9 (16.1)
	>=5	9 (39.1)	14 (42.4)	23 (41.1)
	(Missing)	13 (56.5)	11 (33.3)	24 (42.9)
LDH at start of ICI	Mean (SD)	367.8 (326.1)	250.5 (114.8)	292.2 (218.8)
	Median (IQR)	214.0 (182.8–––455.2)	211.0 (180.0–––281.0)	211.0 (180.0–––305.0)
LDH at start of RT	Mean (SD)	319.4 (311.2)	263.3 (160.6)	282.5 (221.1)
	Median (IQR)	229.0 (199.0 to 285.0)	220.0 (188.0 to 282.0)	221.5 (189.2 to 284.2)
relative PBL count	Mean (SD)	12.4 (4.8)	20.2 (10.0)	17.8 (9.4)
	Median (IQR)	12.1 (9.1 to 14.8)	22.6 (11.6 to 25.5)	16.8 (10.4 to 24.0)
relative PBL count	low	8 (34.8)	8 (24.2)	16 (28.6)
	normal	1 (4.3)	12 (36.4)	13 (23.2)
	(Missing)	14 (60.9)	13 (39.4)	27 (48.2)
mGPS	0	4 (17.4)	13 (39.4)	17 (30.4)
	1	0 (0.0)	3 (9.1)	3 (5.4)
	2	1 (4.3)	5 (15.2)	6 (10.7)
	(Missing)	18 (78.3)	12 (36.4)	30 (53.6)

Abbreviations: BMI: Body Mass Index; UICC: Union Internationale Contre le Cancer; ECOG: Eastern Cancer Oncology Group; TPS: PD-L1 Total Positivity Score; ICI: Immune Checkpoint Inhibition; RT: Radiotherapy; PBL: Peripheral Blood Lymphocytes; mGPS: modified Glasgow Prognostic Score; AP: Abscopal progression; AR: Abscopal response; AC: Abscopal control.

### Radiotherapy

Within the study cohort, 92% of patients (52/56) underwent a single RT course targeting one metastatic site. The majority of patients were treated with hypofractionated (30/56, 54%) or stereotactic RT (22/56, 39%), while a small proportion (7%, 4/56) received normofractionated RT. The most frequently irradiated sites were lung (28.5%, 16/56), bone (26.5%, 15/56), and brain (21.5%, 12/56). The mean total dose ± SD administered was 37 ± 14 Gy, with a median single-fraction dose of 3.75 Gy (IQR: 3–––7.5 Gy). The median planning target volume (PTV) was 192 cc (IQR: 58–––609 cc). Further RT details can be found in Table 2.

**Table 2:** Radiotherapy details.

Label	Levels	AP & Other (no benefit) (N = 23)	AR, AC & min. 1AR (benefit) (N = 33)	Total
PTV (cc)	Mean (SD)	441.2 (508.2)	501.1 (819.9)	476.1 (701.4)
	Median (IQR)	239.2 (59.7 to 711.2)	175.5 (56.1 to 515.4)	192.0 (58.4 to 609.7)
RT-Fractionation	normofractionated	2 (8.7)	2 (6.1)	4 (7.1)
	hypofractionated	12 (52.2)	18 (54.5)	30 (53.6)
	stereotactic	9 (39.1)	13 (39.4)	22 (39.3)
Total RT dose	Mean (SD)	37.8 (14.5)	37.8 (13.8)	37.8 (14.0)
	Median (IQR)	36.0 (30.0 to 41.0)	36.0 (30.0 to 48.0)	36.0 (30.0 to 45.8)
Dose per fraction	Mean (SD)	5.2 (3.9)	5.8 (4.8)	5.5 (4.4)
	Median (IQR)	3.0 (3.0 to 7.0)	4.0 (3.0 to 7.5)	3.8 (3.0 to 7.5)
RT Location	Adrenal gland	4 (17.4)	3 (9.1)	7 (12.5)
	Bone	7 (30.4)	8 (24.2)	15 (26.8)
	Brain	4 (17.4)	8 (24.2)	12 (21.4)
	Liver	1 (4.3)	1 (3.0)	2 (3.6)
	Lung	5 (21.7)	11 (33.3)	16 (28.6)
	Lymphatic system	2 (8.7)	2 (6.1)	4 (7.1)
Ablative RT	Ablative RT	6 (26.1)	12 (36.4)	18 (32.1)
	Not ablative	17 (73.9)	21 (63.6)	38 (67.9)
BED_10_	Mean (SD)	56.7 (26.0)	59.4 (29.5)	58.3 (27.9)
	Median (IQR)	46.8 (43.2 to 58.4)	50.7 (39.0 to 66.3)	47.6 (39.0 to 61.2)
EQD2_10_	Mean (SD)	47.1 (21.8)	49.5 (24.5)	48.5 (23.3)
	Median (IQR)	39.0 (34.2 to 48.6)	42.2 (32.5 to 55.2)	39.7 (32.5 to 51.0)
Dose per fraction at least 5 Gy	No	14 (60.9)	20 (60.6)	34 (60.7)
	Yes	9 (39.1)	13 (39.4)	22 (39.3)
Immunogenic_RT	No	15 (65.2)	22 (66.7)	37 (66.1)
	Yes	8 (34.8)	11 (33.3)	19 (33.9)

Abbreviations; PTV: Planning Target Volume; RT: radiotherapy; Gy: Gray; BED: Biologically effective dose; EQD2: Equivalent dose in 2 Gy fractions; ICI: Immune checkpoint inhibition; AP: Abscopal progression; AR: Abscopal response; AC: Abscopal control. Ablative RT was defined as a total dose of at least 50 Gy EQD2_10_.

### Total non-irradiated tumor burden

We evaluated a total of 139 lesions. Radiological findings from the imaging before RT were compared to tumor volumes at the final imaging assessment of each patient. In two lesions, there was insufficient imaging at the final imaging assessment, resulting in 137 evaluable lesions. As individual patients could have multiple NILs, both a patient-based (n = 56) and a lesion-based (n = 137) analysis were performed, with the lesion-based analysis assessing size reduction on a per-lesion basis, while the patient-based analysis evaluated the AR by looking at all NILs of a patient.

At the individual patient level, we found an AR of all NILs in 16.1% of patients (9/56). In 32.1% (18/56) of patients, we observed AC, while 5.4% (3/56) had AP in all lesions. 10.7% (6/56) showed AR in at least one NIL. In 35.7% of patients (20/56), there was a mixed response with AP in at least one NIL (“other”).

**Fig. 1:**
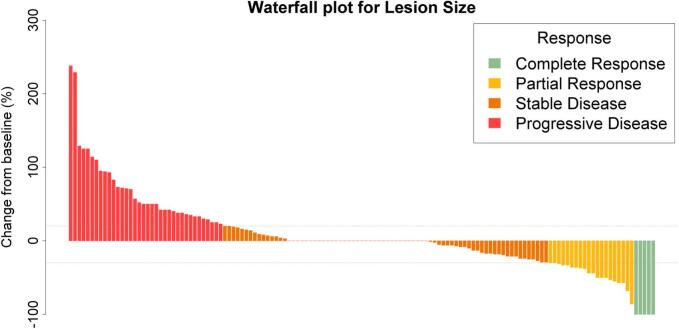
Waterfall plot for each of the 137 evaluated NILs. Most NILs showed stable lesion size. Out of these, many lesions did not demonstrate any change from baseline (stable disease, orange). (For interpretation of the references to colour in this figure legend, the reader is referred to the web version of this article.)

In the lesion-based analysis, 18% (25/137) of lesions showed a size reduction of more than 30%, indicating an abscopal response. Out of these, 20% (5/25) showed a complete response. Most lesions (55%, 76/137) showed no notable change in size (Fig. 1).

### Identification of influencing variables for abscopal response

Patients who experienced AB were more likely to be younger at the start of RT (mean age 61.5 years vs. 67.7 years in the no AB group) and had higher relative peripheral blood lymphocyte (PBL) count prior to RT (20.2% vs. 12.4%). In older patients (>70 years), 77% (7/9) had a relative level of PBL below the normal level (less than 20% of white blood cells), compared to 45% (9/20) in the younger patient cohort. The presence of AB differed between men and women, 76% of women had AR, compared with 48% of men (Table 1). In a multivariable logistic regression model, higher age at radiotherapy was significantly associated with increased odds of AP (OR = 1.07; 95%-CI: 1.00–1.14; p = 0.050). Male sex showed a trend towards higher risk of AP but did not reach statistical significance (OR = 3.13; 95%-CI: 0.93–11.86; p = 0.075). Complete results of the multivariable logistic regression are shown in Suppl. Table 1.

### Survival analysis

**Fig. 2:**
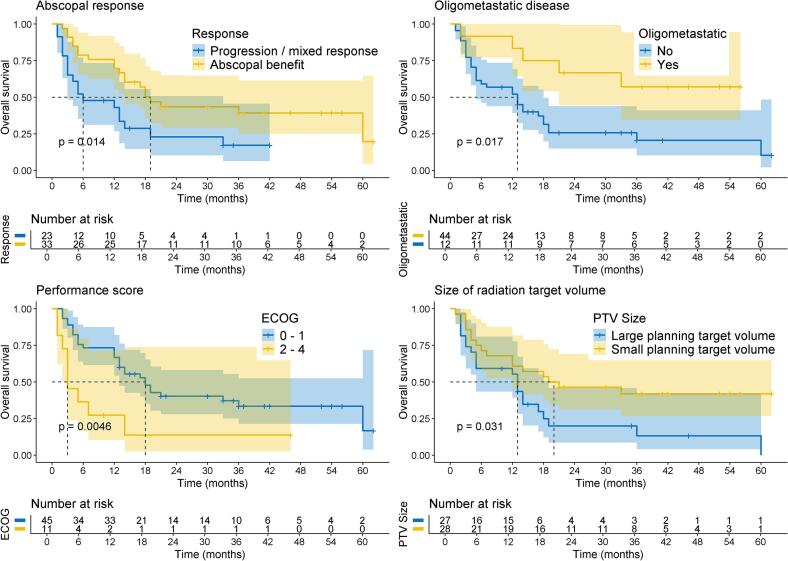
Kaplan-Meier plots for factors that were significantly associated with improved OS in univariable analysis.

The median follow-up was 37 months (95%-CI: 33 – 56 months). The median OS was 14 months (95%-CI: 12 – 33 months). One- and two-year OS was 59% (95%-CI: 47 – 73%) and 35% (95%-CI: 24 – 50%), respectively. The median PFS was 4 months (95%-CI: 3 – 7 months). One- and two-year PFS were 27% (95%-CI: 17 – 41%) and 18% (95%-CI: 10 – 33%), respectively. Factors significantly associated with better OS in univariable log-rank tests were abscopal benefit, oligometastatic disease, good ECOG performance status (ECOG 0–1), and smaller PTV (all p < 0.05, Fig. 2). Anatomical site of irradiated lesions was also associated with OS, although patient numbers per irradiated site were low (p < 0.05, Suppl. Fig. 1).

**Fig. 3:**
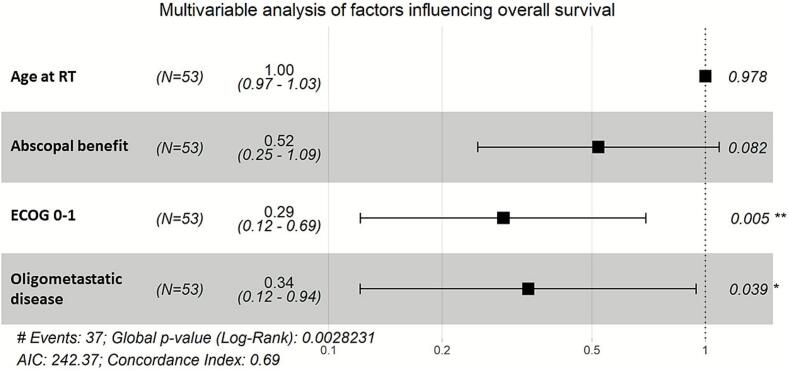
Forest plot of the multivariable analysis for OS. ECOG and oligometastatic disease were significantly associated with OS, while abscopal benefit showed a trend towards improved OS.

The final Cox proportional hazards model for OS included the factors age at RT, AB, oligometastatic disease, and ECOG (Suppl. Table 2A). Here, better performance status (ECOG 0–1) (HR = 0.29; 95%-CI: 0.12–0.69; p < 0.001) and oligometastatic disease (HR = 0.31; 95%-CI: 0.12–0.94; p = 0.039) were associated with better survival outcomes. The presence of AB showed a trend toward improved OS but did not reach statistical significance (HR = 0.52; 95%-CI: 0.25–1.09; p = 0.082). Age at RT was not associated with OS (HR = 1.00; 95%-CI: 0.97–1.03; p = 0.998) (Fig. 3). After landmarking at final imaging to mitigate immortal time bias, AB remained significant in univariable analysis (Suppl. Fig. 2) but did not enter the multivariable model in forward selection (Suppl. Table 2B).

## Discussion

We retrospectively investigated the occurrence rate of AbE in NSCLC patients and evaluated variables that may be associated. In 26.8% of patients, we observed an AR, either in all or in at least one NIL. An AB, meaning at least stable disease in all NILs with or without AR in any NIL, was observed less frequently in men and in older patients. A multivariable survival analysis showed an independent association of better ECOG performance scores and oligometastatic disease stage with OS. The results described in our study are in line with previous reports, which have described an AR rate of 22% for NSCLC treated with radioimmunotherapy [31].

In this study, patients received RT either to continue treatment beyond progression in case of oligoprogressive disease or for the treatment of symptomatic lesions. Treatment failure during ICI is mostly caused by either immunosuppression or immune evasion. These mechanisms are facilitated primarily by the release of pro-tumorigenic factors, such as TGF-β or VEGF, increased expression of inhibitory immune checkpoint molecules, including PD-L1, and by manipulation of the immune system, which favors regulatory immune cells [32]. RT can counter this tumoral immune escape by inducing necrotic and apoptotic release of tumor antigens, aiding in the priming and activation of immune cells and resulting in increased tumor infiltration by cytotoxic T-lymphocytes [33], [34], [35]. Additionally, RT alters the tumor microenvironment in different ways, leading to an enhanced antitumor immune response [36], [37].

Here, we observed two factors associated with AR, namely younger age and female sex. We also observed a relevant difference in the count of PBL between the AB and the AP group. However, PBL counts were available only for a limited number of patients, which is why we did not include this factor in our multivariable logistic regression analysis and why its interpretation should be done with caution. Although a direct comparison between tumor-infiltrating lymphocytes and PBL cannot be drawn from this study, it is noticeable that a higher relative count of PBL was observed in the AB group. Other groups also observed improved OS in patients with higher PBL counts [38]. Increased tumor infiltration by cytotoxic lymphocytes after RT is also supported by other studies that could show increased CD8 + -T-cell activation or the same T-cell receptor clones in both tumor-infiltrating lymphocytes and PBL [39], [40]. Additionally, the relative PBL count might also factor into the effect age had on the abscopal response, as published data suggest immune exhaustion with increased age [41], [42]. In our cohort, 77% of the elderly patients had a lower-than-normal relative count of PBL compared to 45% of the non-elderly patients.

Observation of increased AB based on sex is also in line with other studies focused on the effect of sex on ICI response. There are significant differences in the tumor microenvironment between men and women [43], [44]. While men tend to exhibit T-cell-excluded phenotypes (immune evasion), women have higher intratumoral T-cell infiltration, but also a higher expression of inhibitory immune checkpoint molecules and exhausted T-cell phenotypes (immune suppression) [45], [46].

Our survival analysis is in line with expectations, as ECOG performance status has been shown to be an independent factor for OS in NSCLC [47]. Similarly, oligometastatic disease compared to polymetastatic disease was also associated with improved OS. This has been shown previously in an institutional database analysis [48], although no polymetastatic comparison was made. Other prospective trials have demonstrated high survival rates in selected oligometastatic NSCLC patients [49].

Although the sample size remains relatively small, even after screening a large number of patients, this is – to our knowledge – one of the most extensive retrospective studies focused on AbE in NSCLC [50]. Similarly, in other retrospective studies, authors also reported a high number of screened patients progressing during ICI with only a small number receiving concurrent RT, demonstrating the more recent trend of local treatment for oligoprogressive lesions [50], [51]. For example, in a retrospective study from Utrecht, out of 361 patients with malignant melanoma or NSCLC treated with ICI, only 5 NSCLC patients were treated for extracranial oligoprogression. We prioritized a very robust analysis due to the strict inclusion criteria (no other systemic treatment during the assessed time, ICI before RT, sufficient number of images at fixed time points). This included imaging of NILs between the start of ICI and RT to rule out false-positive responses. To our knowledge, this is the first study in NSCLC patients to integrate such imaging data into its inclusion criteria.

There already exist a few prospective trials evaluating RT in progressive NSCLC patients during ICI treatment. Although they are not focused on the analysis of AR, several AbEs were observed. In a pooled analysis of two prospective trials, Popp et al. demonstrated that RT was able to delay a switch in treatment in progressive NSCLC [52]. However, non-target lesions could have received a significant amount of radiation, which makes the study difficult to interpret in regards to AbE. By implementing very strict inclusion criteria, our study only included the analysis of NILs outside of the 10%-isodose.

The CURB trial also reported results for oligoprogressive NSCLC that received systemic therapy with or without stereotactic body radiotherapy (SBRT) [53]. Here, systemic therapy combined with SBRT resulted in significantly less progression in pre-existing lesions (26%), compared to systemic therapy only (68%). Still, only 35% of patients in the CURB trial had stable disease after RT, as 39% of patients treated with concurrent SBRT developed new lesions. Another recent study reported on a posteriori analysis of patients from the PEMBRO-RT study, wherein 76 patients with advanced NSCLC were randomized and treated either with SBRT followed by anti-PD-1 therapy (n = 35) or anti-PD-1 therapy alone (n = 37) [10], [54]. Here, Huang et al. were able to demonstrate in a detailed molecular analysis of NILs that SBRT prior to ICI was able to induce systemic immunostimulatory effects in immunological cold tumors especially. The authors observed a significant increase in the T cell response in NILs. Additionally, there was an induction of B-cell responses limited to some patients exclusively within the SBRT arm. These patients had durable clinical responses, suggesting the possibility of humoral responses with long-term antitumor immunity.

Some limitations of our study should be mentioned. Due to the retrospective nature, some laboratory values were missing, and the analysis of possible prognostic blood markers is only exploratory and hypothesis-generating. We have elected to include only patients with PD-1- and anti-PD-1-antibodies. Whilst this can be seen as a strength as it leads to a more homogeneous cohort, it is also a limitation as there is a debate that the optimal schedule actually depends on the type of ICI, to which we cannot contribute with this study [55], [56]. Other trials, such as the above-mentioned PEMBRO-RT trial or the ongoing RAD-IO trial (NCT05401786), will be able to give important insights into the optimal sequencing of RT and ICI [10]. In addition, although strict imaging criteria were applied for patient inclusion, it cannot be completely excluded that a small proportion of patients classified as having progressive disease may in fact have experienced pseudoprogression or a delayed response to ICI. However, such events are considered relatively uncommon, and pseudoprogression typically occurs within the first month of ICI treatment [57]. We further accounted for this with our strict inclusion criteria such as using standardized time points for radiological imaging. Consequently, the likelihood that our results were substantially affected by these phenomena can be considered low. In conclusion, this study presents one of the largest retrospective cohorts evaluating AbE in NSCLC patients. RT was able to induce AR in 26.8% of NSCLC patients who were progressive under ICI, possibly delaying a switch in systemic treatment. With local ablative treatment strategies in oligoprogressive NSCLC becoming increasingly common, the occurrence of AbE will be observed more frequently in the future. The results of this study can help shape future prospective trials on AbE in NSCLC patients.

## Declaration of Competing Interest

The authors declare that they have no known competing financial interests or personal relationships that could have appeared to influence the work reported in this paper.
