# Supplementary figures and images for: Radiotherapy in patients with NSCLC developing progressive disease during immune checkpoint inhibition: Abscopal responses and survival
Source: Clin Transl Radiat Oncol. 2026 Feb 11;58:101125. doi: 10.1016/j.ctro.2026.101125 (PMC12973703; doi:10.1016/j.ctro.2026.101125)

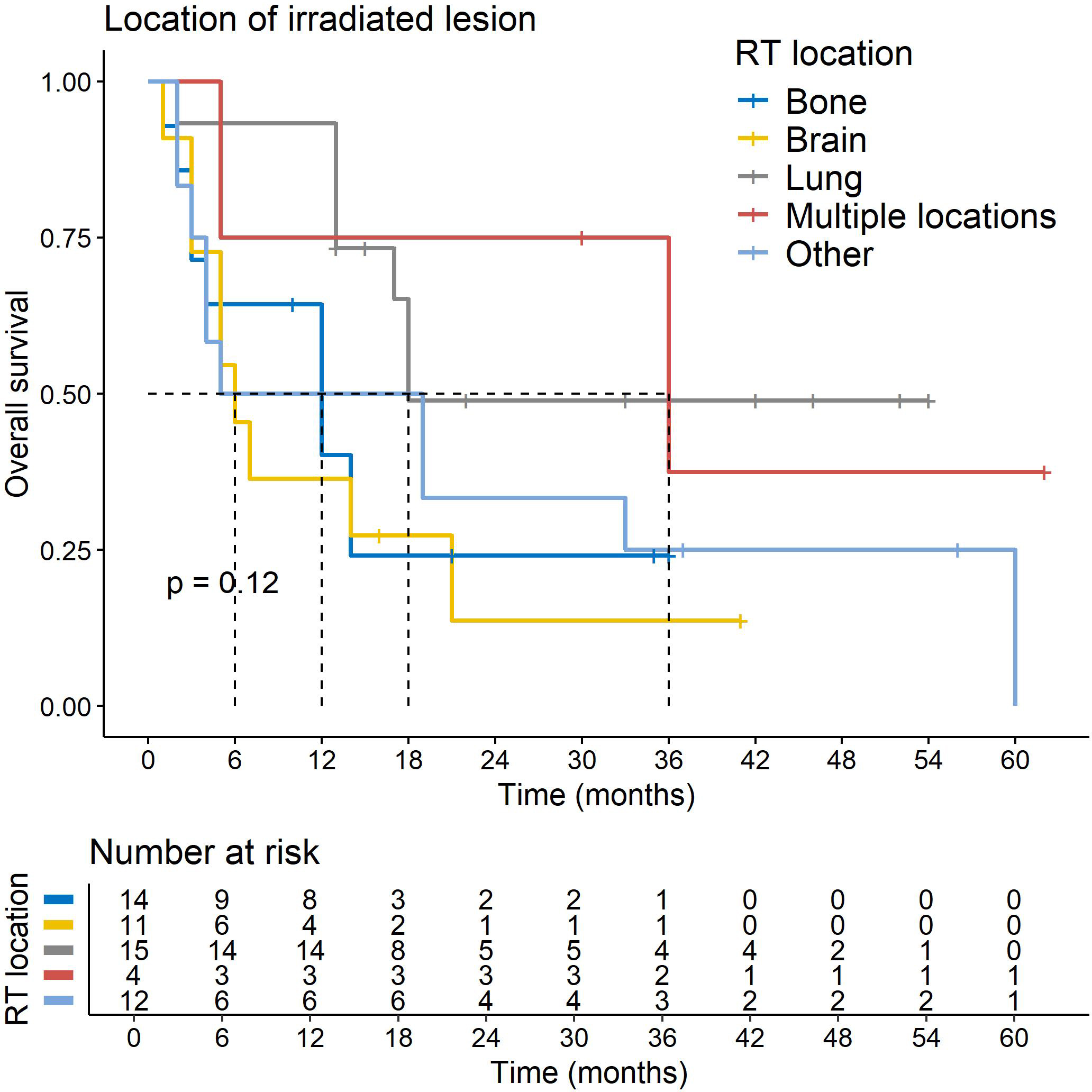

Supplement: Supplementary Fig. 1 [file mmc1.jpg]

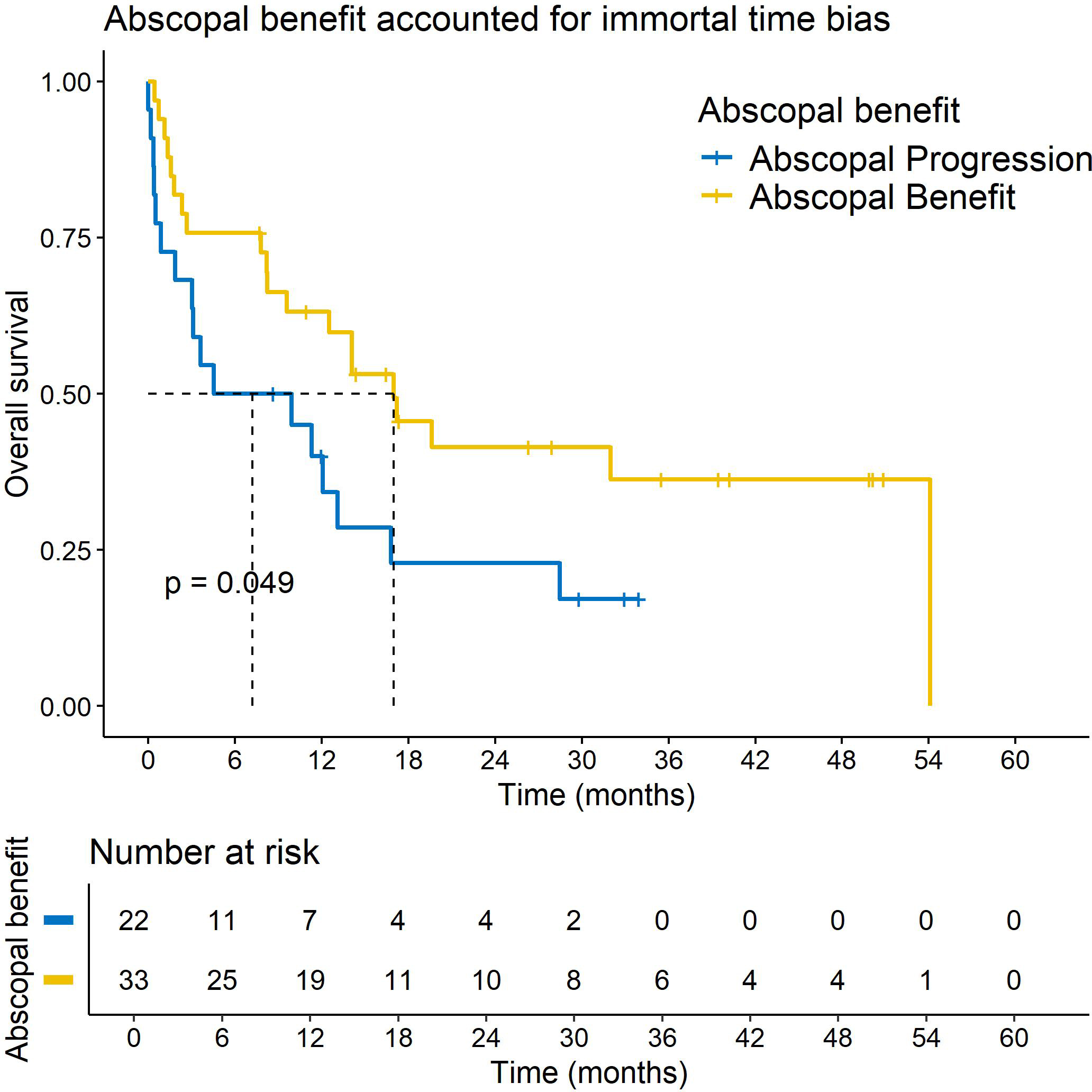

Supplement: Supplementary Fig. 2 [file mmc2.jpg]
