# Supplementary material for: Radiotherapy in patients with NSCLC developing progressive disease during immune checkpoint inhibition: Abscopal responses and survival
Source: Clin Transl Radiat Oncol. 2026 Feb 11;58:101125. doi: 10.1016/j.ctro.2026.101125 (PMC12973703; doi:10.1016/j.ctro.2026.101125)
Supplement: Supplementary Data 1 [file mmc3.docx]

## Suppl. Table 1

| **Predictor** | **B (SE)** | **OR** | **95% CI OR** | **z** | **p** |
| --- | --- | --- | --- | --- | --- |
| Intercept | -5.22 (2.20) | 0.01 | 0.00 – 0.28 | -2.37 | .018 |
| Age at RT (years) | 0.06 (0.03) | 1.07 | 1.00 – 1.14 | 1.96 | .050 |
| Sex (male) | 1.14 (0.64) | 3.13 | 0.93 – 11.86 | 1.78 | .075 |

*Note.* B = unstandardized coefficient (log-odds), SE = standard error, OR = odds ratio, CI = confidence interval, RT = radiotherapy. *p* < .05 indicates statistical significance, but should be considered exploratory.

Multivariable logistic regression on factors associated with abscopal benefit.

## Suppl. Table 2

**A**

| **Predictor** | **B** | **SE** | ***z*** | **HR** | **95% CI for HR** | ***p*** |
| --- | --- | --- | --- | --- | --- | --- |
| Age at RT (years) | –0.0005 | 0.0167 | –0.03 | 1.00 | [0.97, 1.03] | .978 |
| Abscopal benefit | –0.6544 | 0.3764 | –1.74 | 0.52 | [0.25, 1.09] | .082 |
| ECOG 0–1 vs ≥2 | –1.2358 | 0.4448 | –2.78 | 0.29 | [0.12, 0.69] | .005 |
| Oligometastatic | –1.0833 | 0.5236 | –2.07 | 0.34 | [0.12, 0.94] | .039 |

**B**

| **Predictor** | **B** | **SE** | ***z*** | **HR** | **95% CI for HR** | ***p*** |
| --- | --- | --- | --- | --- | --- | --- |
| Age at RT (years) | –0.0069 | 0.0166 | –0.42 | 0.99 | [0.96, 1.03] | .678 |
| Oligometastatic | –1.3776 | 0.5157 | –2.67 | 0.25 | [0.09, 0.69] | .008 |
| ECOG 0–1 vs ≥2 | –1.4088 | 0.4506 | –3.13 | 0.24 | [0.10, 0.59] | .002 |

**Note.** B = regression coefficient; SE = standard error; z = Wald statistic; HR = hazard ratio; CI = confidence interval, RT = radiotherapy. p < .05 indicates statistical significance, but should be considered exploratory.

(A) Multivariable analysis for OS counting from the end of RT (B) Multivariable analysis for adjusted OS to account for immortal time bias.
